# Farnesoid X Receptor Deficiency Induces Hepatic Lipid and Glucose Metabolism Disorder via Regulation of Pyruvate Dehydrogenase Kinase 4

**DOI:** 10.1155/2022/3589525

**Published:** 2022-02-24

**Authors:** Wenyi Deng, Wenjing Fan, Tingting Tang, Hengquan Wan, Simin Zhao, Yao Tan, Kwabena Agyare Oware, Jieqiong Tan, Jiequn Li, Shunlin Qu

**Affiliations:** ^1^Institute of Cardiovascular Disease, Key Laboratory for Arteriosclerology of Hunan Province, University of South China, Hengyang, Hunan, China 421001; ^2^Institute of Clinical Medicine, The Second Affiliated Hospital of Hainan Medical University, Haikou, Hainan, China 570100; ^3^Emergency Department, The Second Affiliated Hospital, University of South China, Hengyang City, Hunan, China 421001; ^4^Center for Medical Genetics and Hunan Key Laboratory of Medical Genetics, School of Life Science, Central South University, Changsha, Hunan, China 410078; ^5^Department of Liver Transplant, Second Xiangya Hospital, Central South University, Changsha, Hunan, China 410011; ^6^Transplant Medical Research Center, Second Xiangya Hospital, Central South University, Changsha, Hunan, China 410011

## Abstract

Farnesoid X receptors (FXR) are bile acid receptors that play roles in lipid, glucose, and energy homeostasis. Synthetic FXR-specific agonists have been developed for treating nonalcoholic fatty liver disease (NAFLD) patients. However, the detailed mechanism remains unclear. To investigate the effects of FXR on NAFLD and the possible mechanism, FXR-null mice were fed either a normal or a high-fat diet. The FXR-null mice developed hepatomegaly, steatosis, accumulation of lipid droplets in liver cells, glucose metabolism disorder, and elevated serum lipid levels. Transcriptomic results showed increased expression of key lipid synthesis and glucose metabolism-related proteins. We focused on pyruvate dehydrogenase kinase 4 (PDK4), a key enzyme involved in the regulation of glucose and fatty acid (FA) metabolism and homeostasis. Subsequently, we confirmed an increase in PDK4 expression in FXR knockout cells. Moreover, inhibition of PDK4 expression alleviated lipid accumulation in hepatocytes caused by FXR deficiency *in vivo* and *in vitro*. Our results identify FXR as a nuclear transcription factor that regulates glucose and lipid metabolism balance through PDK4, providing further insights into the mechanism of FXR agonists in the treatment of metabolic diseases.

## 1. Introduction

Nonalcoholic fatty liver disease (NAFLD) is one of the most common causes of chronic liver disease in Western countries [1]. NAFLD can progress to nonalcoholic steatohepatitis (NASH), liver fibrosis, and even cirrhosis caused by oxidative stress and lipid peroxidation, which may eventually lead to hepatocellular carcinoma, portal hypertension, and liver failure [2–4]. Moreover, NAFLD is associated with a high risk of multiorgan insulin resistance, type 2 diabetes, dyslipidemia, and coronary heart disease [5]. Thus, mechanistic understanding of NAFLD pathogenesis is crucial for developing a therapeutic strategy.

The hallmark feature of NAFLD is steatosis and excessive intrahepatic triglyceride (TG) in the liver of NAFLD patients [6, 7]. The mechanisms of excessive intrahepatic TG include [8] (1) increased supply of free fatty acids (FFAs), including increased lipolysis in both visceral/subcutaneous adipose tissue and/or increased uptake of dietary fat; (2) increased *de novo* hepatic lipogenesis (DNL); (3) decreased FFA oxidation; and (4) reduced removal of intrahepatic TG due to decreased very low-density lipoprotein (VLDL) secretion. FFA delivery to the liver accounts for almost two-thirds of its lipid accumulation [9]. Therefore, elevated peripheral blood FAs and DNL are the main causes of liver fat accumulation in NAFLD [8]. Several rate-limiting enzymes and transcription factors participate in hepatic lipid metabolism. The activation of DNL to TG synthesis in the liver involves sterol regulatory element-binding protein-1c (SREBP-1c) and lipogenic enzymes, including fatty acid synthase (FASN), acetyl-CoA carboxylase 1 (ACC), stearoyl-CoA desaturase-1 (SCD1), and glycerol-3-phosphate acyltransferase 1 (Gpat1) [10]. The expression levels of these enzymes in hepatocytes are significantly upregulated regardless of whether it is in obese humans or rodents, suggesting that an increase in DNL plays a crucial role in hepatosteatosis [11].

Transcription factor functions play a central role in regulating lipid metabolism. Farnesoid X receptor (FXR) is one of the most important bile acid and nuclear receptor in the liver and is highly expressed in the liver and intestines [12]. Activation of FXR apparently regulates bile acid synthesis in the liver, its transport within the enterohepatic circulation, and a series of physiological processes [13–16]. Growing evidence suggests that the FXR signaling pathway is involved in lipid metabolism, glucose homeostasis, and in the pathogenesis and progression of NAFLD [17]. Efforts has been made to utilize bile acid, a small molecule with strong physiological effects, and FXR agonists for therapeutic applications [13]. Obeticholic acid (OCA) is the most promising synthetic FXR agonists that has been approved for the treatment of primary biliary cholangitis [18]. OCA has been shown to increase insulin sensitivity in patients with type 2 diabetes and to improve markers of liver inflammation and fibrosis [19]. The effectiveness of OCA therapy has also been demonstrated in a multicentre trial of patients with noncirrhotic, nonalcoholic steatohepatitis [20]. The improvement of histological features of NASH by OCA may be related to the activation of FXR, which reduces hepatic lipogenesis by downregulating transcription factor SREBP1c and increasing Sirtuin 1 (SIRT1) [21, 22]. However, the exact mechanism underlying the effect of the bile acid signaling pathway on energy metabolism is unclear.

In this study, we found that FXR null (FXR^−/−^) mice fed either with a normal or a high-fat diet were hepatomegaly with lipid droplet aggregation and elevated plasma lipids. We demonstrated that FXR regulated liver lipid droplet accumulation and IR by mediating pyruvate dehydrogenase kinase 4 (PDK4) gene expression. These results expand understanding of FXR regulatory mechanisms of lipid metabolism and promote the clinical application of FXR agonists.

## 2. Materials and Methods

### 2.1. Reagents and Antibodies

Rabbit polyclonal antibodies against FASN (#3180), PGC-1*α*(#2178), SCD1(#2438), ACLY(#4332), p-p38 MAPK(#4511), ACC(#4190), P-AMPK*α*1 (#2537), p-mTOR-S244–8(#5536), mTOR(#2983), and *α*-tubulin(#2144) were from Cell Signaling Technology. Anti-PDK4(#DF7169) antibodies were from Affinity Biosciences. Anti-GCK(#ab137714), anti-CD36 (#ab133625), and anti-PDK4(#ab89295) antibodies were from Abcam. Anti-NR1H4(#PP-A9033A-00) was from R&D Systems. Anti-SREBP-1c (#Sc-13551) was purchased from Santa Cruz. Sodium dichloroacetate (#347795) was purchased from Sigma-Aldrich. Sodium oleate/sodium palmitate (#SYSJKJ006) was purchased from Xi'an Kun Chuang Technology Company. All other chemicals were from Sigma-Aldrich.

### 2.2. Animals

The animal experimental protocol was conformed to the Animal Ethics Committee of the University of South China and followed the approved guidelines. Eighty 1-3-month-old male FXR knockout C57BL/6 mice (FXR^−/−^) and C57BL/6 wild type (wt) mice were obtained from Jackson Laboratory. Mice were housed in SPF facilities. To determine the effect of FXR on glucose and lipid metabolism, mice with matched body weight were randomized for four groups and then either maintained on the standard chow diet (SD,13% fat, 3.66 Kcal/g, #1010009, Xietong, Nanjing, China) or received a high-fat diet (HFD, 60% lard fat, 5.13 Kcal/g, #XTHF60, Jiangsu Xietong Pharmaceutical Bio-engineering Co., Ltd. Nanjing, China) for 12 weeks; randomization was accomplished as follows: SD-WT (*n* = 10), SD-FXR^−/−^(*n* = 10), HFD-WT (*n* = 10), and HFD-FXR^−/−^(*n* = 10). The body weight of the mice and the food consumed by mice were weighed every 3 days. After 71 days, the mice were subjected to a glucose tolerance test (GTT), and all mice were subjected to an insulin tolerance test (ITT) after 80 days. The animals were sacrificed 12 weeks later. Venous blood, livers, and adipose tissue (VAT) depots from inguinal subcutaneous (SAT) and visceral were isolated, rinsed with 0.9% NaCl, snap-frozen in liquid nitrogen, and stored at -80°C for further analysis.

To determine the role of PDK4 in the process of FXR regulating glucose and lipid metabolism, mice were randomized into the following four groups: HFD-WT (*n* = 10), HFD-FXR^−/−^(*n* = 10), HFD-FXR^−/−^+low-DCA [intraperitoneal injection 100 mg/kg/day dichloroacetate (DCA, *n* = 10)], and HFD-FXR^−/−^+high-DCA [intraperitoneal injection 300 mg/kg/day dichloroacetate (DCA, *n* = 10)]. The body weight of the mice and the food consumed by mice were weighed every 3 days. After 49 days, the mice were subjected to a GTT, all mice were subjected to an ITT after 54 days. The animals were sacrificed immediately 8 weeks later. Venous blood, livers, and adipose tissue (VAT) depots from inguinal subcutaneous (SAT) and visceral were isolated, rinsed with 0.9% NaCl, snap-frozen in liquid nitrogen, and stored at -80°C for further analysis.

### 2.3. Histology

Liver and adipose tissues from the mice were fixed in phosphate-buffered 4% paraformaldehyde. The right lateral lobule of the liver was then divided into 2 sections at the long middle line, one of which was embedded in paraffin blocks and the other in the O.C.T. compound. Samples were frozen and sectioned at a thickness of 25 *μ*m with a Leica cryostat. Lipid droplets were determined by Oil Red O staining. Take 25 *μ*m paraffin section for morphology with hematoxylin and eosin (H&E); the relative content of PDK4 expression was detected by immunohistochemistry.

### 2.4. Intraperitoneal Glucose Tolerance Test

The intraperitoneal glucose tolerance tests (IPGTT) were performed after fasting for 12 hours, and then, glucose (2 g/mL) was injected into them intraperitoneally. Blood samples were collected from the tail vein, and blood glucose was measured at 0 (before the glucose injection), 15, 30, 60, 90, and 120 min after the glucose injection using a blood glucose meter (ROCHE, Germany).

### 2.5. Intraperitoneal Insulin Tolerance Test

The intraperitoneal insulin tolerance test (IPITT) was performed five days after the IPGTT test. The mice were fasted for 6 h and then intraperitoneally injected with insulin (1 U/kg). Blood samples were collected from the tail vein, and blood glucose was measured at 0 (before the insulin injection), 15, 30, 60, 90, and 120 min after the insulin injection using a blood glucose meter (ROCHE, Germany).

### 2.6. Serum Analyses

Total blood samples were also collected for measurement of fasting serum total cholesterol (TC), triglyceride (TG), and nonesterified fatty acid (NEFA). Determination of blood lipids and liver enzyme index in serum was conducted by the automatic biochemical analyzer (JEOL Ltd, Japan).

### 2.7. Intracellular and Liver Triglyceride Measurement

Intracellular and liver triglycerides were assayed using a triglyceride assay kit (#E1013, Applygen Technologies Inc., China) according to the manufacturer's recommended protocol. Intracellular and hepatic free fatty acids (FFA) were estimated using an ultrasensitive assay kit for free fatty acids (#BC0595, Beijing Solar bio Science &Technology Co., Ltd., China) according to the manufacturer's recommended protocol.

### 2.8. Cell Line

L-02 cells (human hepatic cell line) were obtained from the Type Culture Collection of the Chinese Academy of Sciences (Shanghai, China). FXR-null L-02 cells were generated by the CRISPR-Cas9 system, as described [23]. The targeting sgRNA sequences (*NR1H4*: 5′-TCCCTGCTGACGCGCCC ATG-3′) were subcloned into LentiCRISPRv2 (#49535, Addgene, USA). Recombinant LentiCRISPR plasmid was cotransfected with pCMV-VSV-G (#8454, Addgene, USA) and psPAX2 (#12260, Addgene, USA) into HEK293T cells to package infectious lentivirus. Medium-containing viruses were used to infect cells for 8 h and then selected in 0.5 *μ*g/mL puromycin. Genomic DNA was isolated and amplified by PCR (forward: 5′-TTTGTTTTAGGCTTGTTAAC-3′, Reverse: 5′-TTGGACTAG AAATTCAGCTG-3′) followed by Sanger sequencing. Two independent FXR-null L-02 clones were selected for further analysis.

### 2.9. Cell Culture and Treatment

L-02 WT cells and FXR-null L-02 cells were cultured in 1640 medium (Gibco, USA) containing 10% fetal bovine serum (Gibco, USA) and 1% penicillin/streptomycin (Invitrogen, USA) in a humidified atmosphere of 5% CO_2_ at 37°C, and fresh medium was changed daily. When the cell confluence reached 30%, nontargeted control or targeted PDK4 siRNAs (5′-CTACTCGATGCTGATGAA-3′) were transfected into FXR-null L-02 using Lipofectamine 3000 Reagent (#L3000-015, Invitrogen, USA). Cells were incubated with 1640 complete medium supplemented with 500 *μ*M/250 *μ*M sodium palmitate/sodium oleate (SYSJKJ006, China) for 6 h, then the medium was replaced with the flash medium. After 6 h of culture, the cells were washed twice with PBS. Finally, BODIPY493/503 (#D3922, Thermo Fisher Scientific, USA) staining was performed to check the lipid droplet formation.

### 2.10. RNA-Seq Analysis

Total RNA was isolated using TRIzol® Reagent (Thermo Fisher Scientific, USA) following the manufacturer's protocol and cDNA library generation with the TruSeq RNA Sample Preparation Kit (Illumina, USA). Clusters were generated with the TruSeq SR Cluster Kit v2 according to the reagent preparation guide. The RNA sequencing was performed using the Illumina platform. High-quality reads were aligned to the mouse reference genome (mm9) using the SOAP aligner. The expression levels for each of the genes were normalized to reads per kilobase of exon model per million mapped reads (RPKM) to compare mRNA levels between samples. The genes associated with glucose and lipids metabolism were retrieved from the Cufflinks output for further analysis.

### 2.11. Western Blot Analysis

The liver tissues were homogenized in 2× SDS sample buffer (63 mM Tris-HCl, 10% glycerol, and 2% SDS containing protease inhibitor and phosphatase inhibitor). The cultured cells were washed twice with 1× PBS and finally added the appropriate amount of 2× SDS sample buffer for lysis. The protein concentration of samples was detected using a BCA Protein Assay Kit (23225, Thermo Fisher Scientific, USA). 20 *μ*g protein were separated by SDS polyacrylamide gels and transferred onto polyvinylidene fluoride membranes, followed by immunoblotting with specific antibodies. Membranes were then incubated with a peroxidase-conjugated secondary antibody. Specific bands were detected with a Bio-Rad Imaging System (Bio-Rad, USA).

### 2.12. Real-Time PCR

Total RNA from liver tissue was extracted using TRIzol® Reagent (Thermo Fisher Scientific, USA) and was reverse transcribed into cDNA with the RevertAid RT Kit (Thermo Fisher Scientific, USA) according to the manufacturer's instruction. SYBR Green qPCR Master Mix (#K0251, Thermo Fisher Scientific, USA) was used for quantitative real-time PCR amplification with a CFX96 real-time PCR detection system (Bio-Rad, USA) and corresponding software.

### 2.13. Statistical Analysis

Statistical analysis was performed using Prism 7 software (GraphPad Software, USA). The data were reported as the means ± standard error. A two-tailed Student's *t*-test was used to determine the difference between the two groups. Analysis of differences between groups was performed using one-way ANOVA and Tukey. Quantitation was performed by double-blinded experimenters. *P* < 0.05, the difference was statistically significant.

## 3. Results

### 3.1. FXR Deficiency Causes Hepatomegaly, Body Weight Loss, Blood Glucose Metabolism Disorder, and Elevated Serum Lipids in Mice

**Figure 1:**
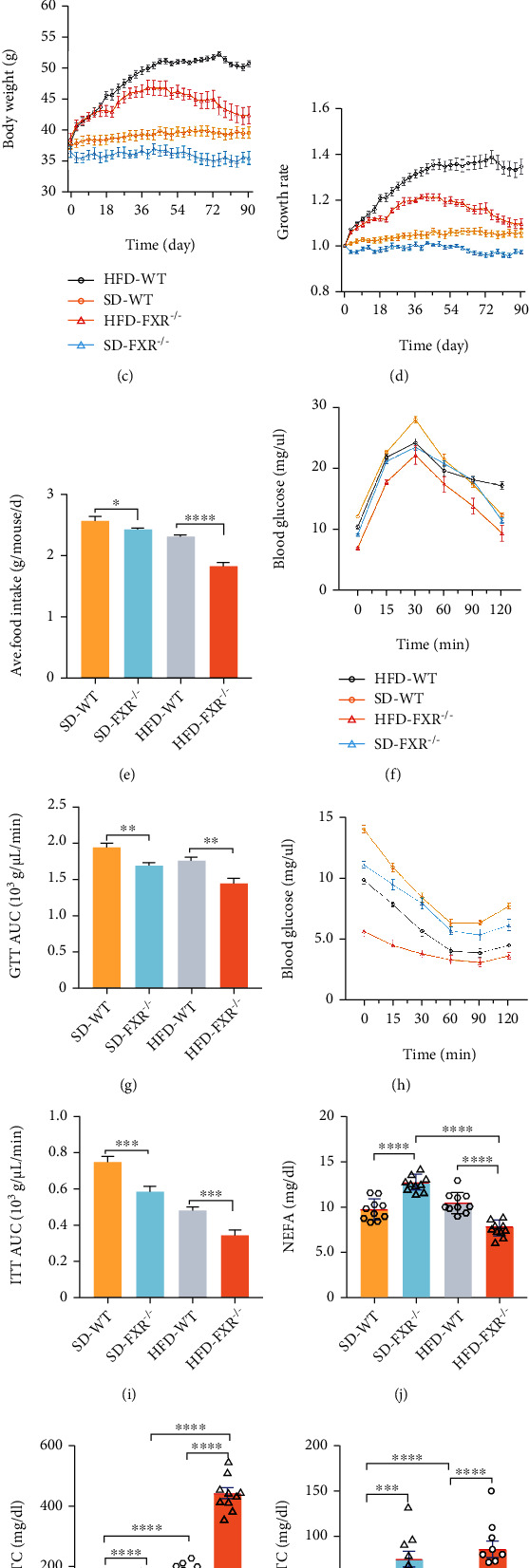
FXR deficiency caused liver hepatomegaly, dyslipidemia, and glucose metabolism disorder in mice. (a, b) Hepatomegaly of FXR-null mice. Liver tissue control (WT) and FXR null (knockout [KO]) mice (a) and quantitative analysis of liver tissue mass related to their body weight are shown (b). Standard chow diet (SD) and high-fat diet (HFD) (*n* = 10). (c, d) Decreased bodyweight of FXR-null mice. Body weight changes in mice fed a normal diet and high-fat diet for three months were recorded, and their growth rates were calculated. (e) Decreased food intake of FXR-null mice (*n* = 10). (f, g) High-fat diet and FXR deficiency caused glucose tolerance in mice. After 71 days of feeding the mice, the mice were subjected to a glucose tolerance test (*n* = 10). (h, i) High-fat diet and FXR deficiency caused insulin tolerance in mice. After the 80th day, the mice were subjected to an insulin sensitivity test (*n* = 10). (j–l) Elevated serum lipids in FXR-null mice. NEFA, TC, and TG content in FXR-null mice and WT fed with SD and HFD diet. ^∗^*P* < 0.05, ^∗∗^*P* < 0.01, ^∗∗∗^*P* < 0.001, ^∗∗∗∗^*P* < 0.0001 (*n* = 10).

To investigate the pathophysiology of FXR, we examined FXR-null mice at 3, 6, 9, and 12 months. At age 3 months, the FXR-null mice show generally normal. At age 6 months, FXR-null mice showed a gradual loss of body weight compared to the wild-type (WT) littermates (Figure S1A). Histological analysis revealed an increase in intracellular vacuolation of liver tissue in 6-month-old FXR-null mice (Figure S1B). However, abdominal and subcutaneous adipose tissue was not affected (Figure S1C, D). 3-month-old FXR-null mice fed on normal and high-fat diet for 90 days also showed hepatomegaly, uneven pleats, and a slightly greasy cut surface on the liver compared to the WT littermates (Figures 1(a) and 1(b)). Interestingly, after 5 weeks, the body weight gain of mice fed on high-fat diet showed a decrease (Figures 1(c) and 1(d)). Food intake was also reduced in FXR-null mice after a period of high-fat feeding (Figure 1(e)).

GTT and ITT showed significant changes in plasma glucose or insulin levels, as evident from the area under the curve (AUC) of glucose in the FXR-null mice compared to the WT littermates (Figures 1(f)–1(i)). Serum NEFA, TC, and TG levels were higher in FXR-null mice as compared to the WT littermates (Figures 1(j)–1(l)). Taken together, these results indicate that the FXR deficiency causes abnormal glucose and lipid metabolism in mice.

### 3.2. FXR Deficiency Leads to Increased Lipid Deposition, Forming Lipid Vacuoles, Increased Expression of Key Lipid Synthesis Proteins, and Glucose Metabolism-Related Proteins in Mice

**Figure 2:**
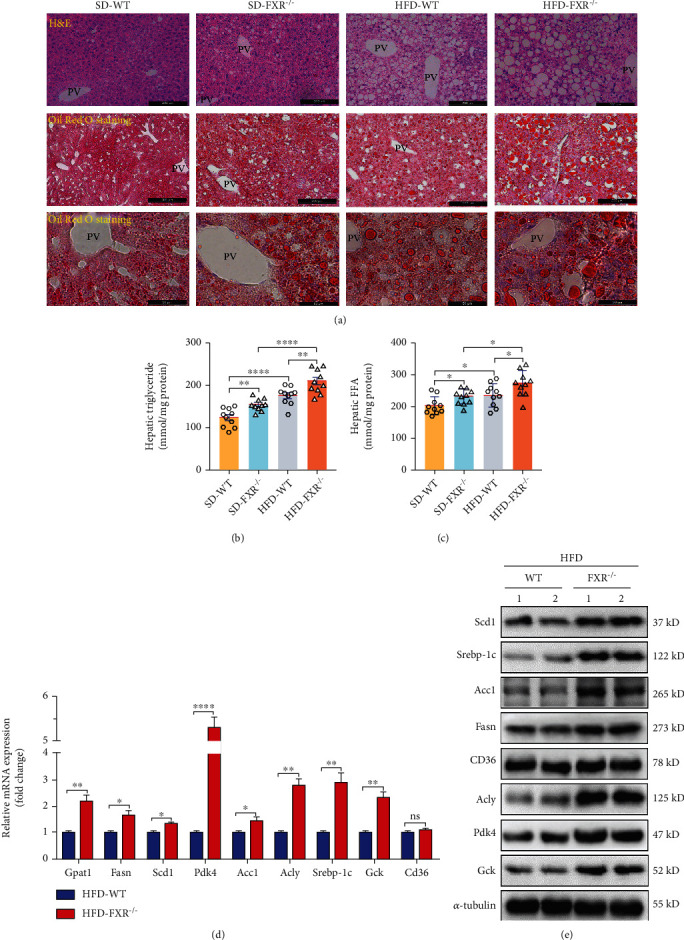
FXR deficiency increased hepatocyte lipid deposition and glucose metabolism and accelerated deterioration of hepatic steatosis in FXR-null mice. (a) A high-fat diet and FXR deficiency increased hepatocyte lipid deposition. PV: portal vein. Upper panel: bar, 200 *μ*m, representative images H&E staining of liver tissues; middle panel: bar, 200 *μ*m, lower panel: bar, 50 *μ*m; both of them representative images of Oil Red O staining of liver tissues; WT: *n* = 3, KO: *n* = 3. SD: standard chow diet, HFD: high-fat diet. (b, c) FXR deficiency increased hepatic TG and FFA level, *n* = 10. (d) Relative mRNA levels of PDK4 and relevant glycolytic and lipogenic genes in the livers of FXR-null and WT control mice deal with the high-fat diet, HFD-WT: *n* = 3, HFD-KO: *n* = 3. (e) Detection expression of Scd1, PDK4, Srebp-1c, Acly, Acc1,CD36, Fasn, Gck, and tubulin. ^∗^*P* < 0.05, ^∗∗^*P* < 0.01, ^∗∗∗∗^*P* < 0.0001.

Histological analysis revealed increased intracellular vacuolation in liver tissues and accumulation of Oil Red O stain-positive lipid droplets (Figure 2(a)), which was accompanied by increased levels of liver TG and FFA content in FXR-null mice compare with the WT littermates (Figures 2(b) and 2(c)). High-fat feeding aggravates intrahepatic lipid droplet aggregation and increases the content of TG and FFA (Figures 2(a)–2(c)). Furthermore, the mRNA and protein level of genes related to lipogenesis and glucose metabolism, including glycerol-3-phosphate acyltransferase 1 (*gpat1*), stearoyl-CoA desaturase 1 (*scd1*), sterol regulatory element-binding protein 1c (*srebp-1c*), FA synthase (*fasn*), acetyl-CoA carboxylase 1 (*acc1*), ATP-citrate lyase (*acly*), glucokinase (*gck*), and *pdk4* were also increased in FXR-null mice (Figure 2(d)), whereas cluster of differentiation 36 (*CD36*) mRNA and protein levels were not significantly different (Figures 2(d) and 2(e)). These results indicated that the FXR deficiency increased DNL rather than FA uptake, resulting in hepatosteatosis in mice. Reduced FXR expression in the liver is reported to be responsible for hepatic steatosis in aging mice [24]. Furthermore, consistent with previous studies, Gck expression was increased in the livers of FXR-null mice suggesting that the hepatic Gck levels are associated with liver fat in NAFLD [25]. Together, these results demonstrated that decreased FXR expression causes the impairment of lipid and glucose metabolism in the liver.

### 3.3. The Expression of PDK4 Was Increased in the Liver of FXR-Null L-02 Cells and Mice

**Figure 3:**
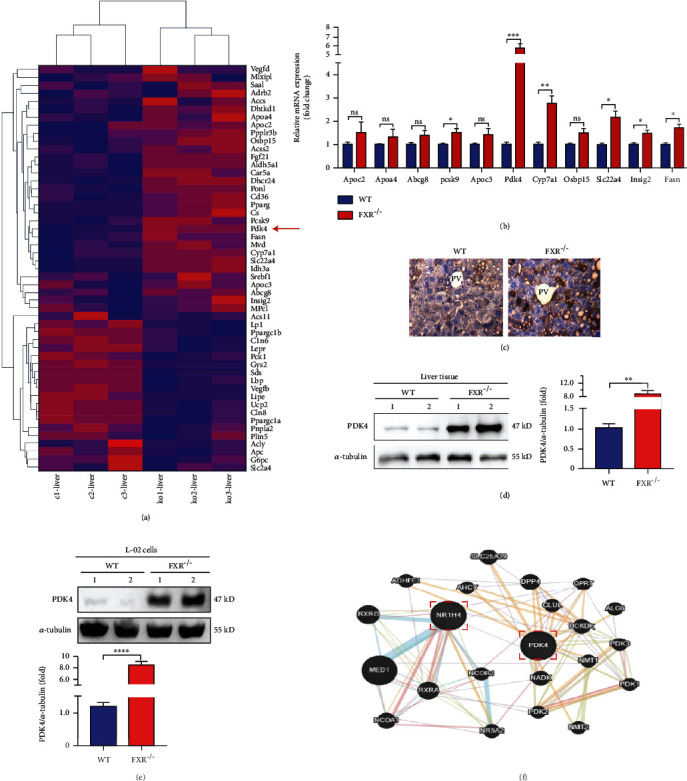
FXR deficiency increased hepatocyte's expression of PDK4. (a) An unbiased RNA-seq expression profiling heat map between liver tissues of WT littermates (c1, c2, and c3) and FXR-null mice (ko1, ko2, and ko3). (b) mRNA levels of PDK4 and genes related to lipid metabolism were detected by qRT-PCR in the liver of FXR-null and WT mice, *n* = 3. (c) Immunohistochemical staining has detected the expression of PDK4 in liver tissue, bar = 50 *μ*m. Representative images of liver tissues of control (WT) and FXR null mice with immunohistochemical staining are shown, *n* = 3. (d) Liver tissue lysates were immunodetected for PDK4 and *α*-tubulin, relative levels of PDK4/*α*-tub are shown, and two independent FXR-null mice (KO1 and 2) were analyzed, *n* = 3. (e) FXR-null L-02 (KO) and control cells (WT) lysates were immunodetected for PDK4 and *α*-tubulin, relative levels of PDK4/*α*-tub are shown, two independent FXR-null L-02 cell lines (KO1 and KO2) were analyzed. (f) Through the predictive website of genetic interactions-Gene MANIA (http://genemania.org), searched for links between FXR and PDK4. ^∗^*P* < 0.05, ^∗∗^*P* < 0.01, ^∗∗∗^*P* < 0.001, ^∗∗∗∗^*P* < 0.0001, *n* = 3.

As a nuclear receptor, FXR is mainly expressed in enterohepatic tissues and is a major regulator of bile acid, lipid, and glucose homeostasis [26]. To identify the FXR-regulated genes, global gene expression in the liver was assessed by RNA-sequencing (RNA-seq) analysis in FXR-null and the WT control mice. The results showed that the genes related to glucose and lipid metabolism were significantly different in FXR-null mouse liver (Figure 3(a)). In particular, the increase of PDK4 was most significant in FXR-null mice (Figures 2(d) and 3(b)). Consistent with the mRNA levels, immunostaining and Western blot results showed that PDK4 was also increased in FXR-null mice compared to the WT littermates livers (Figures 2(e), 3(c), and 3(d)). PDK4 plays a key role in the regulation of glucose and fatty acid metabolism and homeostasis via phosphorylation of the pyruvate dehydrogenase subunits (PDHA1 and PDHA2) [27]. PDK4 expression is elevated in human NASH liver specimens, and deletion of PDK4 can alleviate nonalcoholic fatty liver in mice [28, 29]. The specific mechanism of PDK4 in lipid accumulation in the liver in FXR-null mice received our interest. We also generated FXR-null L-02 cells using the CRISPR/Cas9 system (Figure S2A, B). Western blot analysis revealed that FXR-deficient human hepatocytes also caused an increase in PDK4 expression (Figure 3(e)). We also used GeneMANIA (http://genemania.org) to determine the possible relationships between the FXR and PDK4. The results showed that FXR (NR1H4) and PDK4 were directly related (Figure 3(f)). Thus, the data suggest that hepatic lipid metabolism disorder caused by FXR deficiency may be associated with increased PDK4 expression.

### 3.4. PDK4 Inhibition Alleviated Lipid Accumulation in Hepatocytes Caused by FXR Deficiency In Vivo

**Figure 4:**
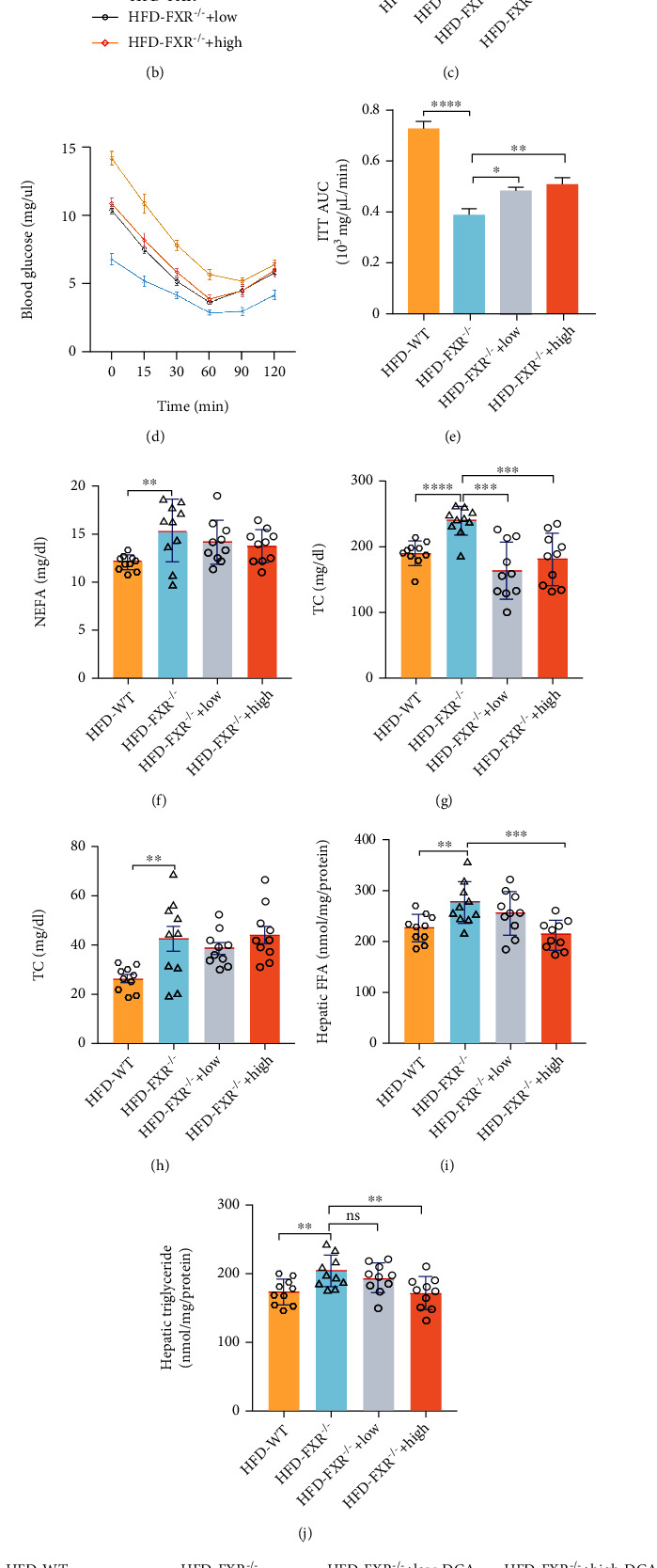
DCA relieved hepatomegaly and reduced liver lipid droplet accumulation in FXR-null mice. Mice were divided into WT mice-saline group, FXR-null mice-saline group, FXR-null mice-100 mg/kg/day (low) DCA group, FXR-null mice-300 mg/kg/day (high) DCA group. All the mice were fed a high-fat diet for two months. (a) Experimental protocol of the in vivo study. (b, c) DCA alleviated glucose tolerance in FXR-null mice. After 49 days, the mice were subjected to a glucose tolerance test, *n* = 10. (d, e) DCA enhanced insulin sensitivity in FXR-null mice. After the 54th day, the mice were subjected to an insulin sensitivity test. *n* = 10. (f–h) DCA reduced blood lipids in FXR-null mice. NEFA, TC, and TG content in FXR-null mice with DCA treatment, *n* = 10. (i, j) DCA treatment reduced hepatic TG and FFA level, *n* = 10. (k) DCA reduced hepatocytes lipid deposition. PV: portal vein. Upper panel: bar, 200 *μ*m, representative images H&E staining of liver tissues; middle panel: bar, 200 *μ*m, lower panel: bar, 50 *μ*m; both of them representative images of Oil Red O staining of liver tissues; WT: *n* = 3, KO: *n* = 3. ^∗^*P* < 0.05, ^∗∗^*P* < 0.01, ^∗∗∗^*P* < 0.001, ^∗∗∗∗^*P* < 0.0001.

To investigate the role of PDK4 in lipid accumulation in the liver caused by the FXR deficiency, we determined lipid accumulation in FXR-null mice after inhibiting PDK4. Dichloroacetate (DCA) is a mitochondrial PDK inhibitor that activates pyruvate dehydrogenase (PDH) by inhibiting PDK dephosphorylation; this results in a large amount of acetyl CoA that enters the mitochondria to initiate the citric acid cycle for promoting oxidative phosphorylation of glucose [30]. DCA showed no effect on food intake and body weight in FXR-null mice (Figure S3A-C). However, DCA alleviated hepatomegaly and abdominal fat production caused by a high-fat diet in FXR-null mice (Figure S3D, E). The GTT and ITT showed glucose sensitivity significant improvement; DCA treatment increased the glucose or insulin levels in a dose-dependent manner, as evident from the AUC of glucose compared with FXR-null mice (Figures 4(b)–4(e)). These results showed that DCA ameliorated abnormal glucose metabolism caused by FXR deficiency. We also found that DCA treatment had no effect on TG in the serum but reduced the levels of TC and FFA in the serum of FXR-null mice fed a high-fat diet (Figures 4(f)–4(h)). Histological analysis revealed that DCA also reduced the accumulation of lipid droplets, the contents of hepatic FFA and TG (Figures 4(i) and 4(j)), and hepatic vacuolation (Figure 4(k)). Together, the data suggest that PDK4 plays a critical role in hepatic steatosis induced by a high-fat diet in FXR-null mice.

### 3.5. DCA Suppressed Fatty Acid Synthesis Enzymes and Altered Multiple Hepatic Signaling Pathways in FXR-Null Mice

**Figure 5:**
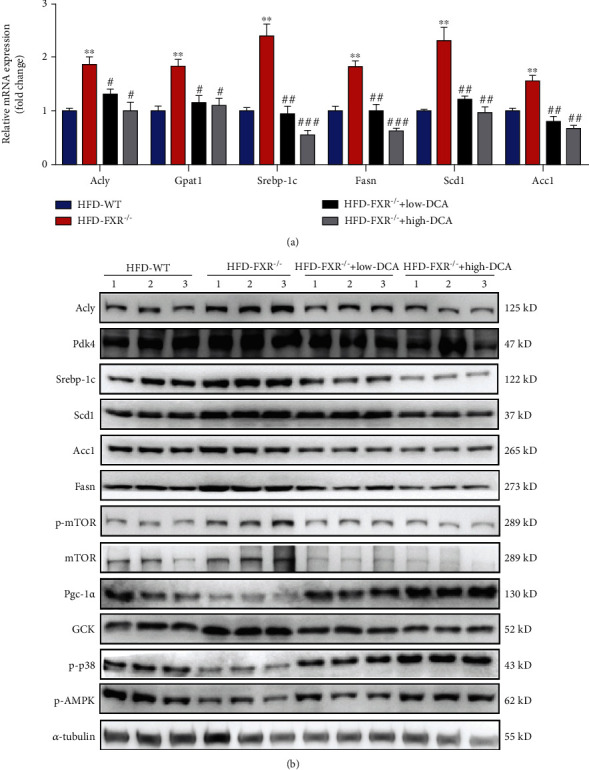
Effect PDK inhibitor (dichloroacetate) on expression of enzymes involved in lipid metabolism, gluconeogenesis, and multiple hepatic signaling pathways. (a) Relative mRNA levels of PDK4 and relevant glycolytic and lipogenic genes in the livers of FXR-null, wild-type mice fed high fat diet with DCA treatment. ^∗∗^*P* < 0.01, relative to wild-type mice fed high fat diet, ^#^*P* < 0.05, ^##^*P* < 0.01, ^###^*P* < 0.001, relative to FXR-null mice fed high-fat diet with DCA treatment, *n* = 3. (b) Representative Western blots are shown for the amounts of Fasn, Srebp-1c, Scd1, Acc1, PGC-1*α*, mTOR/p-mTOR, p-AMPK, p-p38, PDK4, Acly, and GCK in the livers of wild-type or FXR-null mice fed the high-fat diet with DCA treatment. Tubulin served as the loading control.

To understand the mechanism of PDK4 regulation lipid metabolism, the expression of enzymes involved in FA synthesis was measured by qRT-PCR and Western blotting. The expression of Fasn, Srebp-1c, Scd1, and Acc1 was increased in FXR-null mice compared to WT littermates, whereas this increase was reversed in a dose-dependent manner after treatment with PDK4 inhibitor DCA (Figure 5(a)). The FXR deficiency decreased the expression of peroxisome proliferator-activated receptor-*γ* coactivator-1*α* (Pgc-1*α*), a transcription factor that promotes the expression of enzymes involved in FA oxidation. The reduction in the expression of this factor in FXR-null mice was attenuated by DCA. Acly is an important enzyme linking glucose catabolism to lipogenesis by providing acetyl-CoA from the mitochondrial citrate for FA and cholesterol biosynthesis [31]. Acly-deficient hepatocytes protect against hepatic steatosis and dyslipidemia [32]. We found that the deficiency of FXR led to increased expression of Acly in liver tissue, while DCA inhibited the high expression of Acly (Figure 5(b)).

We evaluated major signaling pathways reported to be related to NAFLD pathology. Adenosine 5′-monophosphate- (AMP-) activated protein kinase (AMPK) is a central regulator of energy balance. AMPK phosphorylates specific enzymes and growth control nodes to increase ATP generation and decrease ATP consumption [33]. Reduced AMPK phosphorylation was observed in FXR-null mice compared with the WT littermates. However, after DCA treatment, reduced AMPK phosphorylation was restored. p38*α* plays an important role in glucose homeostasis and lipid metabolism [34]. Concerning lipid metabolism, liver-specific p38*α* knockout mice were more susceptible to high-fed diet-induced obesity and steatosis accompanied by reduced FA *β*-oxidation [35]. Our results clearly showed that the deletion of FXR decreased the phosphorylation of p38*α*, and the inhibitor of PDK restored the phosphorylation level of p38*α*. The mTOR signaling pathway was also reported to promote *de novo* lipogenesis through the activation of SREBP1. The PDK inhibitor DCA also reduced the phosphorylation level of mTOR caused by FXR deficiency (Figure 5(b)).

### 3.6. PDK4 Interference Decreased Lipid Deposition in FXR-KO L-02 Cells

**Figure 6:**
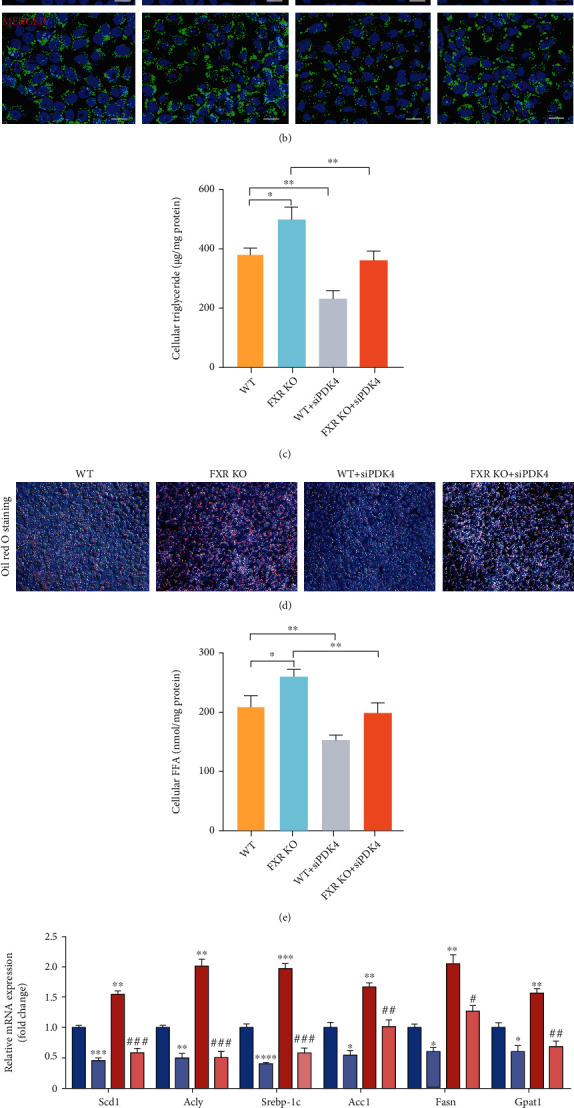
PDK4 interference reduced lipid synthesis in FXR-null L-02 cells (a) Quantization of fluorescence intensity. *n* = 3. (b) BODIPY staining and BODIPY493/503 were labeled lipid droplets in cells. Fluorescence intensity was detected. Amplification factor: 10 × 40, green represents lipid droplets. (c, e) Influence of PDK4 interference on intracellular TG and FFA level induced by sodium oleate/sodium palmitate, *n* = 3. (d) Oil Red O staining of cells treated with sodium oleate/sodium palmitate after transfection of siRNAPDK4 or negative siRNA. Original magnification, 10 × 20. (f, g) Relative mRNA levels of relevant lipogenic genes in FXR-null L-02 cells by pretreatment with siRNAPDK4. ^∗^*P* < 0.05, ^∗∗^*P* < 0.01, ^∗∗∗∗^*P* < 0.0001, *n* = 3.

To explore the mechanism of FXR in hepatic cells *in vitro*, we constructed the FXR knockout (KO) L-02 cell line using CRISPR/Cas9 gene-editing technology (Figure S2A, B). The expression of PDK4 was increased in the FXR KO cell line compared to that in the control cells (WT). We performed BODIPY dye and Oil Red O staining to detect the accumulation of lipid droplets in FXR KO L-02 cells. The results showed that the lipid droplet fluorescence intensity in FXR KO L-02 cells was significantly higher than that in L-02 control cells (Figures 6(a) and 6(b)). Oil Red O staining also showed lipid droplets aggregates in FXR KO L-02 cell lines (Figure 6(d)). Next, we explored whether the accumulation of lipid droplets caused by FXR deletion was related to the increased expression of PDK4. We used siRNA to silence the expression of PDK4 in L-02 cells. BODIPY fluorescent staining results showed that the fluorescence intensity of lipid droplets in FXR KO L-02 cells (150.3 ± 8.819) with PDK4 interference was significantly lower than that in FXR KO L-02 cells (196.7 ± 6.692) (Figures 6(a) and 6(b)). Oil Red O staining results were also consistent with BODIPY fluorescent staining results (Figure 6(d)), suggesting that PDK4 interference inhibits lipid accumulation caused by FXR deficiency. We also detected increased content of TG and FFA in FXR KO cells that were restored after PDK4 interference (Figures 6(c) and 6(e)). To gain insight into the mechanism by which FXR deficiency increases the number of lipid droplets in FXR KO L-02 cells, the protein levels of enzymes involved in FA synthesis were measured. In FXR KO L-02 cells, the amounts of Scd1, Acc1, Fasn, Srebp-1c, and Acly were increased by sodium oleate/sodium palmitate treatment compared to the control cells. However, the amounts of these enzymes with siPDK4 were lower than the amounts present in FXR KO L-02 cells with sodium oleate/sodium palmitate treatment (Figures 6(f) and 6(g)). Consistent with the *in vivo* results, we found that the accumulation of lipid droplets caused by the increase in PDK4 expression in FXR-null hepatic cells may be related to the mTOR signaling pathway; however, elucidation of the detailed mechanism requires further study. The data suggest that PDK4 plays an indispensable role in FXR deficiency-induced lipid droplet accumulation.

## 4. Discussion

Recent studies have suggested that lipid metabolism and glucose homeostasis may be related to nuclear receptor mediation, including FXR, pregnane X receptor (PXR), and constitutive androstane receptor (CAR). Among these nuclear receptors, the FXR signaling pathway is largely involved in lipid metabolism and glucose homeostasis, as well as in the pathogenesis and progression of NAFLD [36, 37]. FXR expression is decreased in nonalcoholic liver disease and diabetes [24, 38, 39]. The major finding of this study is that FXR maintains glycolipid metabolism in hepatocytes by regulating PDK4 expression. The findings of hepatic steatosis and hyperlipidemia in FXR-null mice in the present study were consistent with previously reported results in the literature [40, 41]. Our results suggest a potential therapeutic strategy for treating metabolic syndrome by targeting FXR in the liver. OCA, the most promising synthetic FXR agonist, was recently shown in a randomized, placebo-controlled clinical trial to improve liver histology in patients with NASH [20]. The FXR deficiency resulted in increased lipid content in the liver, serum cholesterol, and TGs in mice, whereas bile acid or synthetic agonist GW4064 reduced liver steatosis and plasma TGs by activating FXR [42, 43].

The main cause of lipid accumulation in the liver is related to the increased activities of key lipid synthetases such as Scd1, Sredp-1c, Fasn, Acc1, and Gpat1 [44, 45]. Our results confirmed that FXR deficiency in mice fed a high-fat diet significantly activated the expression of the lipogenic genes. FXR has been shown to regulate adipogenesis [12, 46]. FXR-deficient mice also showed elevated blood glucose and decreased glucose and insulin tolerance, revealing that FXR also plays an important role in maintaining glucose homeostasis [47]. However, the association between glucose metabolism and adipogenesis is not well understood. Here, we found that the glucose metabolism-related gene PDK4 may mediate abnormal lipid metabolism in FXR-null mice. PDK4 is a mitochondrial enzyme that inhibits the conversion of pyruvate to acetyl-CoA by inhibiting the phosphorylation of the pyruvate dehydrogenase complex (PDC) [48]. Inactivation of PDC by PDKs can inhibit the conversion of pyruvate to acetyl-CoA, resulting in a shift of pyruvate to the citric acid cycle or FA synthesis toward gluconeogenesis [49]. PDK4 deficiency leads to the inhibition of FA oxidation and increases glucose oxidation due to the greater PDC activity, which in turn increases the conversion of pyruvate into acetyl-CoA [48]. Under diabetic conditions, the expression of PDK genes, especially PDK4, is significantly elevated in the liver and leads to an increase in gluconeogenesis; in contrast, PDK4 knockout led to better glucose tolerance suggesting that the hepatic PDK4 may be critically involved in the pathogenesis of diabetes [50]. These observations can help us in explaining the decreased glucose tolerance and insulin sensitivity caused by FXR deficiency, which must be related to the increased expression of PDK4 in the liver. Although we have demonstrated that FXR can affect the expression of PDK4, whether FXR regulates PDK4 at the transcription level remains to be determined.

High-fat-diet FXR-null mice treated with the PDK inhibitor DCA showed improved glycaemic control and glucose tolerance. The predominant site of *de novo* lipogenesis is the liver, and FAs produced are esterified to glycerol to form TGs, packaged with cholesterol esters, cholesterol, phospholipids, and proteins to form very-low-density lipoprotein (VLDL) which are then exported to peripheral tissues [51]. Inactivation of PDC by PDKs can inhibit the conversion of pyruvate to acetyl-CoA, resulting in a shift of pyruvate to the citric acid cycle or FA synthesis toward gluconeogenesis [49]. Our results show that inhibition of PDK4 expression alleviates lipid accumulation in hepatocytes caused due to FXR deficiency *in vivo* and *in vitro*. When DCA was used as an inhibitor of PDK (particularly for PDK4 in the liver), lipid accumulation was reduced in the liver of FXR-null mice and PDK4 expression interference decreased lipid production in FXR KO L-02 cells in *vitro*. Moreover, FXR deficiency led to increased TG and FFAs in both the serum and liver of mice, and PDK4 interference significantly alleviated the abnormal increase in TG and FFAs caused by FXR deficiency *in vitro*.

Mechanistically, our results also showed that PDK4 regulated mTOR, AMPK, PGC-1*α*, P38, and other signaling pathways, all of which are involved in lipid metabolism. The mTOR signaling pathway has been reported that activation of SREBP1 promotes lipogenesis [52]. This is consistent with our results; FXR deficiency activated the mTOR signaling pathway, and DCA reversed these effects. PGC-1*α* is a transcription coactivator required for the expression of genes involved in mitochondrial biogenesis, hepatic gluconeogenesis, and FA oxidation. Knocking out PGC-1*α* induces fat accumulation in the liver [53]. Interference with PDK4 could lead to higher levels of PGC1*α*, consistent with a lower capacity for *de novo* FA synthesis.

In conclusion, the findings of this study provide novel insights into the contribution of PDK4 to hepatic steatosis and illuminate a potential pathogenic mechanism underlying FXR mutant disease.

## Data Availability

The data used to support the findings of this study are included within the article.

## Supplementary Materials

Supplementary 1 Supplementary Table S1: the primer sequences used in quantitative real-time PCR.

Supplementary 2 Figure S1: FXR-null mice develop hepatocellular vacuolation. Figure S2: characterization of the FXR-deficient cell lines. Figure S2: characterization of the FXR-deficient cell lines. Figure S3: the effect of DCA on body weight and on diet in FXR deficient mice.
