# Supplementary material for: Farnesoid X Receptor Deficiency Induces Hepatic Lipid and Glucose Metabolism Disorder via Regulation of Pyruvate Dehydrogenase Kinase 4
Source: Oxid Med Cell Longev. 2022 Feb 24;2022:3589525. doi: 10.1155/2022/3589525 (PMC8896157; doi:10.1155/2022/3589525)
Supplement: Supplementary 1 — Supplementary Table S1: the primer sequences used in quantitative real-time PCR. [file 3589525.f1.docx]

**Supplementary Table S1.** The primer sequences used in quantitative Real-Time PCR.

| Srebp-1c (Human) | Forward, 5’-ACAGTGACTTCCCTGGCCTAT-3’;  Reverse, 5’-GCATGGACGGGTACATCTTCAA-3’ |
| --- | --- |
| Srebp-1c (Mouse) | Forward, 5’-TGACCCGGCTATTCCGTGA-3’;  Reverse, 5’-CTGGGCTGAGCAATACAGTTC-3’ |
| Fasn (Human) | Forward, 5’-AAGGACCTGTCTAGGTTTGATGC-3’;  Reverse, 5’-TGGCTTCATAGGTGACTTCCA-3’ |
| Fasn (Mouse) | Forward, 5’-GGAGGTGGTGATAGCCGGTAT-3’;  Reverse, 5’-GGAGGTGGTGATAGCCGGTAT-3’ |
| Scd1 (Human) | Forward, 5’-TCTAGCTCCTATACCACCACCA-3’;  Reverse, 5’-TCGTCTCCAACTTATCTCCTCC-3’ |
| Scd1 (Mouse) | Forward, 5’-TTCTTGCGATACACTCTGGTGC-3’;  Reverse, 5’-CGGGATTGAATGTTCTTGTCGT-3’ |
| Acc1 (Human) | Forward, 5’-ATGTCTGGCTTGCACCTAGTA-3’;  Reverse, 5’-CCCCAAAGCGAGTAACAAATTCT-3’ |
| Acc1 (Mouse) | Forward, 5’-GATGAACCATCTCCGTTGGC-3’;  Reverse, ﻿5’-ACCCAATTATGAATCGGGAGTG-3’ |
| Gpat1 (Human) | Forward, 5’-GATGTAAGCACACAAGTGAGGA-3’;  Reverse, 5’-TCCGACTCATTAGGCTTTCTTTC-3’ |
| Gpat1 (Mouse) | Forward, 5’-ACAGTTGGCACAATAGACGTTT-3’;  Reverse, 5’-CCTTCCATTTCAGTGTTGCAGA-3’ |
| Acly (Human) | Forward, 5’-ACTTCGGCAGAGGTAGAGCA-3’;  Reverse, 5’-CAGGAGTGACCCGAGCATAC-3’ |
| Acly (Mouse) | Forward, 5’-ACCCTTTCACTGGGGATCACA-3’;  Reverse, 5’-GACAGGGATCAGGATTTCCTTG-3’ |
| Pdk4 (Human) | Forward, 5’-CAAGATGCCTTTGAGTGTTCAA-3’;  Reverse, 5’-GGTCTTCTTTTCCCAAGACAAC-3’ |
| Pdk4 (Mouse) | Forward, 5’-CCATGAGAAGAGCCCAGAAGA-3’;  Reverse, 5’-GAACTTTGACCAGCGTGTCTACAA-3’ |
| Cd36 (Mouse) | Forward, 5’-ATGGGCTGTGATCGGAACTG-3’;  Reverse, 5’-ATGGGCTGTGATCGGAACTG-3’ |
| Gck (Mouse) | Forward, 5’-TCCCTGTAAGGCACGAAGA-3’;  Reverse, 5’-GAGAAGTCCCACGATGTTGTT-3’ |
| GAPDH (Human) | Forward, 5’-CAGAACATCATCCCTGCCTCTAC-3’;  Reverse, 5’-TTGAAGTCAGAGGAGACCACCTG-3’ |
| GAPDH (Mouse) | Forward, 5’-GAGGGTGGAGCCAAAAG-3’;  Reverse, 5’-GCTGACAATCTTGAGTGAGTTG-3’ |
