# Supplementary material for: Farnesoid X Receptor Deficiency Induces Hepatic Lipid and Glucose Metabolism Disorder via Regulation of Pyruvate Dehydrogenase Kinase 4
Source: Oxid Med Cell Longev. 2022 Feb 24;2022:3589525. doi: 10.1155/2022/3589525 (PMC8896157; doi:10.1155/2022/3589525)
Supplement: Supplementary 2 — Figure S1: FXR-null mice develop hepatocellular vacuolation. Figure S2: characterization of the FXR-deficient cell lines. Figure S2: characterization of the FXR-deficient cell lines. Figure S3: the effect of DCA on body weight and on diet in FXR deficient mice. [file 3589525.f2.docx]

**Supplementary materials**

**Figure S1.FXR-null mice develop hepatocellular vacuolation.** control (WT) and FXR-null (knockout [KO]) mice (A) at age 3–12 month and analysis their body weight are shown. *, P < 0.05. WT=10, KO=10. (B) H&E staining of liver tissues. Representative images of liver tissues of control (WT) and FXR null (KO) mice with H&E stain are shown. PV, portal vein. Upper panel: bar, 200 µm; lower panel: bar, 50 µm. (C and D) H&E staining of adipose tissues. Representative images of abdominal(C) and subcutaneous(D) adipose tissue of control (WT) and FXR-null (KO) mice with H&E stain are shown. Bar, 200 µm, n=3.


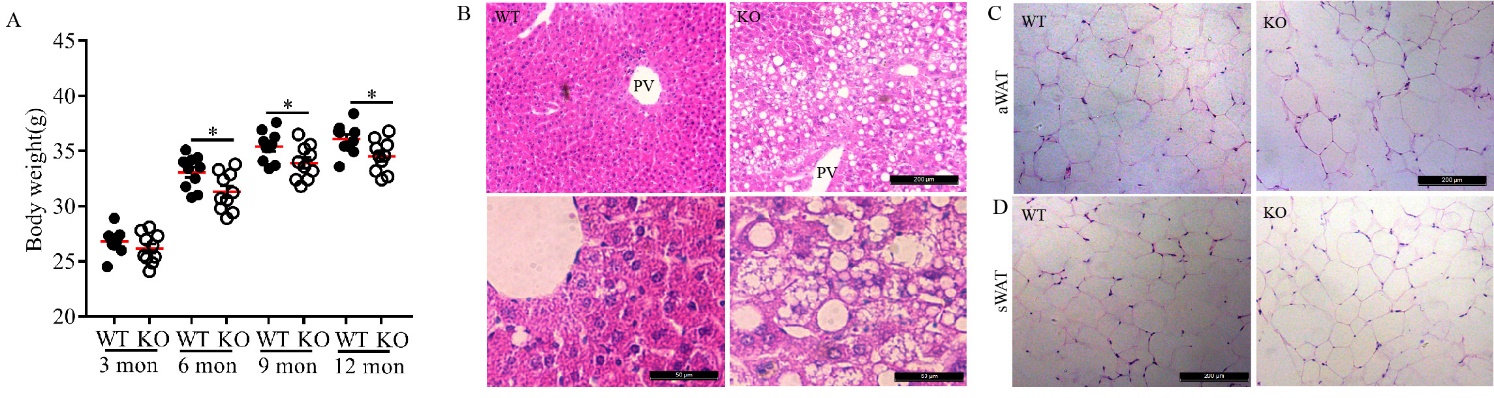


**Figure S2. Characterization of the FXR-deficient cell lines.** Characterization of FXR-null L-02 cells. FXR-null cells were generated with the CRISPR-Cas9 system. (A)Shown are representative sequencing results of FXR-null clones. (B) FXR mutant clones were analyzed by immunoblotting using antibodies against either FXR antibody or β-actin, n=3.


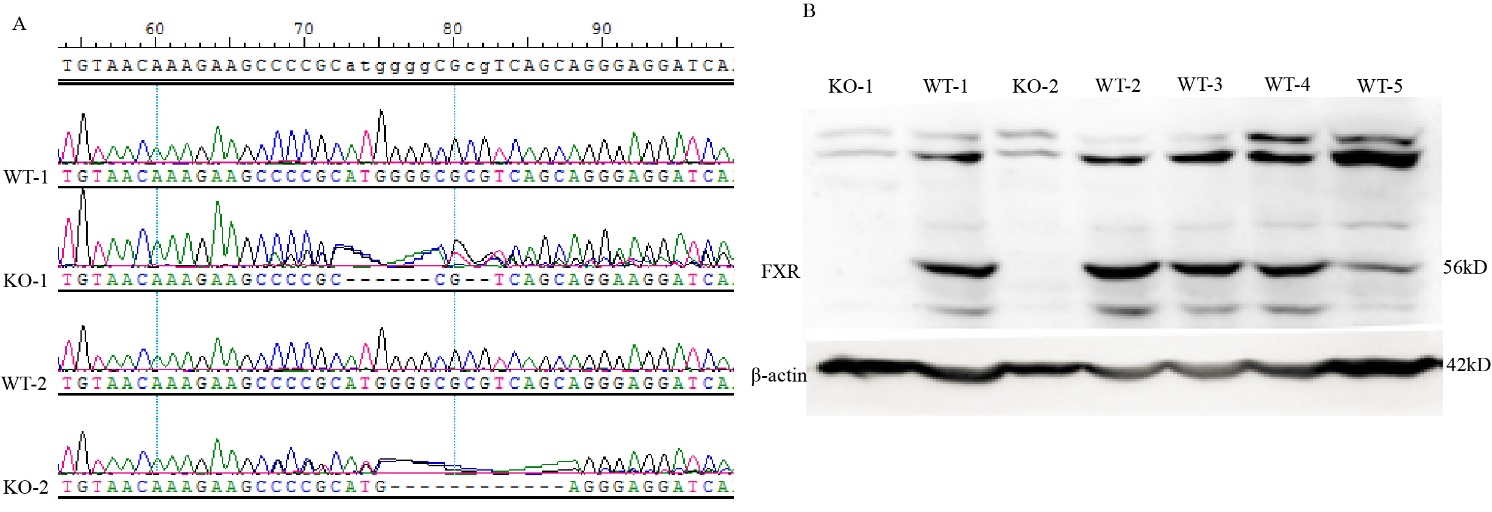


**Figure S3. The effect of DCA on body weight and on diet in FXR deficient mice.** (A) DCA has no effect on body weight in FXR-deficient mice. Body weight changes in mice fed high-fat diet for two months with abdomen injection DCA were recorded, and their growth rates calculated. (B) The effect of DCA on the diet of FXR-deficient mice. **p<0.01, (n=10). (C) quantitative analysis of liver tissue mass related to their body weight are shown. ***, P<0.001, ****, P<0.0001. (D) quantitative analysis of abdominal adipose mass related to their body weight are shown, ****, P<0.0001,n=10.

**
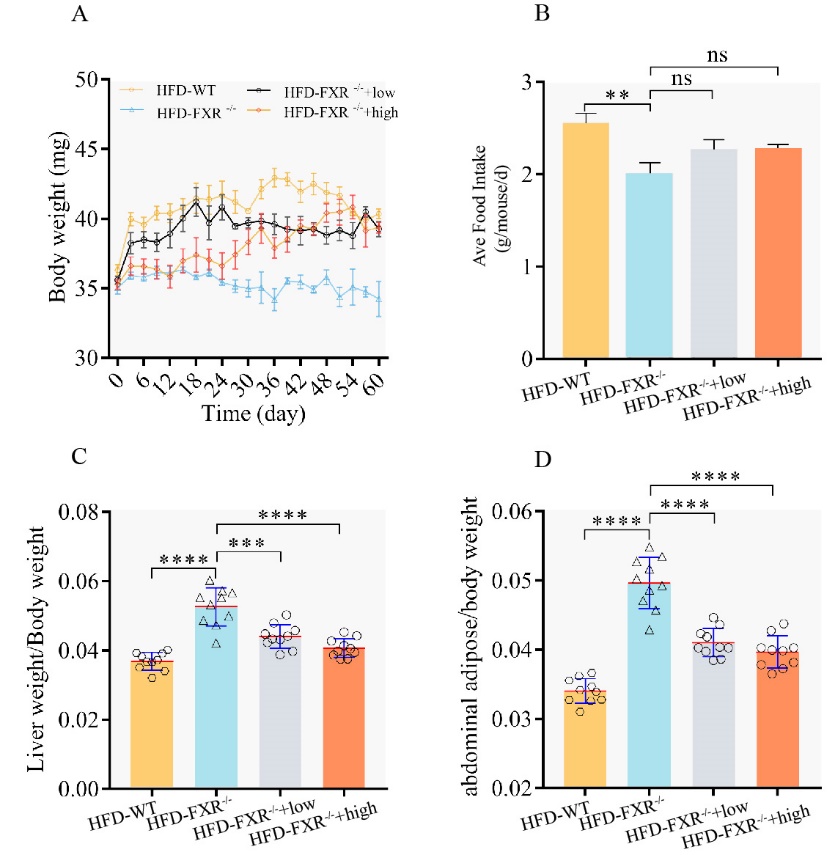
**
